# Quantitative whole-brain 3D imaging of tyrosine hydroxylase-labeled neuron architecture in the mouse MPTP model of Parkinson's disease

**DOI:** 10.1242/dmm.042200

**Published:** 2019-11-22

**Authors:** Urmas Roostalu, Casper B. G. Salinas, Ditte D. Thorbek, Jacob L. Skytte, Katrine Fabricius, Pernille Barkholt, Linu M. John, Vanessa Isabell Jurtz, Lotte Bjerre Knudsen, Jacob Jelsing, Niels Vrang, Henrik H. Hansen, Jacob Hecksher-Sørensen

**Affiliations:** 1 Gubra, Hørsholm Kongevej 11B, 2970 Hørholm, Denmark; 2 Department of Obesity Research, Global Drug Discovery, Novo Nordisk A/S, 2760 Måløv, Denmark; 3 Department of Bioinformatics and Data Mining, Novo Nordisk A/S, 2760 Måløv, Denmark; 4 Department of Diabetes Research, Global Drug Discovery, Novo Nordisk A/S, 2760 Måløv, Denmark

**Keywords:** Imaging, iDISCO, Light-sheet fluorescence microscopy, Tyrosine hydroxylase, Parkinson's disease, Neurotoxicity model

## Abstract

Parkinson's disease (PD) is a basal ganglia movement disorder characterized by progressive degeneration of the nigrostriatal dopaminergic system. Immunohistochemical methods have been widely used for characterization of dopaminergic neuronal injury in animal models of PD, including the MPTP (1-methyl-4-phenyl-1,2,3,6-tetrahydropyridine) mouse model. However, conventional immunohistochemical techniques applied to tissue sections have inherent limitations with respect to loss of 3D resolution, yielding insufficient information on the architecture of the dopaminergic system. To provide a more comprehensive and non-biased map of MPTP-induced changes in central dopaminergic pathways, we used iDISCO immunolabeling, light-sheet fluorescence microscopy (LSFM) and deep-learning computational methods for whole-brain three-dimensional visualization and automated quantitation of tyrosine hydroxylase (TH)-positive neurons in the adult mouse brain. Mice terminated 7 days after acute MPTP administration demonstrated widespread alterations in TH expression. Compared to vehicle controls, MPTP-dosed mice showed a significant loss of TH-positive neurons in the substantia nigra pars compacta and ventral tegmental area. Also, MPTP dosing reduced overall TH signal intensity in basal ganglia nuclei, i.e. the substantia nigra, caudate-putamen, globus pallidus and subthalamic nucleus. In contrast, increased TH signal intensity was predominantly observed in limbic regions, including several subdivisions of the amygdala and hypothalamus. In conclusion, mouse whole-brain 3D imaging is ideal for unbiased automated counting and densitometric analysis of TH-positive cells. The LSFM–deep learning pipeline tracked brain-wide changes in catecholaminergic pathways in the MPTP mouse model of PD, and may be applied for preclinical characterization of compounds targeting dopaminergic neurotransmission.

## Abstract (precis)

**Summary:** Whole-brain immunolabeling, mapping and absolute quantification of tyrosine hydroxylase neurons in the adult mouse brain provides a useful tool for studying changes in dopaminergic signaling in a mouse model of PD.

## INTRODUCTION

The dopaminergic system plays a fundamental role in motor control, cognitive function, motivational behavior, feeding and reward (Klein et al., 2019). Consequently, perturbations in dopaminergic neurotransmission have been implicated in various CNS conditions, including Parkinson's disease (PD) (Obeso et al., 2008), schizophrenia (Grace and Gomes, 2019), and drug and food addiction (Kenny, 2011; Volkow et al., 2017). Although PD neuropathology encompasses a number of different neurotransmitter pathways (Barone, 2010), the cardinal motor manifestations of PD have been attributed primarily to deficits in brain dopamine signaling pathways (Obeso et al., 2008). The classical model of parkinsonism involves reduced nigrostriatal connectivity due to progressive loss of functional projections from dopamine-producing neurons in the substantia nigra pars compacta (SNc). The end result of striatal dopamine depletion is imbalanced excitatory and inhibitory striatal output that propagates throughout the basal ganglia circuits, giving rise to the cardinal motor manifestations of PD, i.e. rigidity, bradykinesia and resting tremor (Kreitzer and Malenka, 2008; Obeso et al., 2008).

Most hypotheses about the etiology and pathogenesis of dopamine deficiency in PD have been derived from animal models. In particular, neurotoxin-based models have provided the opportunity to study the pathophysiological changes occurring in the basal ganglia in association with the parkinsonian state. The 1-methyl-4-phenyl-1,2,3,6-tetrahydropyridine (MPTP) model is one of the most common models applied in preclinical PD research, as administration of MPTP produces reliable and reproducible neurotoxic lesions of the nigrostriatal dopaminergic pathway after systemic administration (Dauer and Przedborski, 2003). To date, MPTP is the only neurotoxin capable of promoting a parkinsonian syndrome in both non-human primates and intoxicated humans, being indistinguishable from the motor disabilities in idiopathic PD (Langston et al., 1983; Potts et al., 2014). Also, the MPTP mouse model shows progressive biochemical and histopathological changes in dopaminergic pathways similar to those observed in MPTP-dosed non-human primates (Jakowec and Petzinger, 2004), and has for decades been the most widely used mouse model in preclinical research on dopamine-associated molecular mechanisms and drug targets in PD (Duty and Jenner, 2011).

Immunohistochemical detection of tyrosine hydroxylase (TH) expression, the rate-limiting enzyme in catecholamine synthesis (Molinoff and Axelrod, 1971), has provided an essential tool for visualizing and quantifying damage and loss of dopaminergic neurons in animal models of PD. Evidently, unbiased estimation of TH-positive (TH+) dopaminergic neuron numbers is crucial for phenotyping and histological assessment of treatment effects. Stereological methods based on the optical fractionator method and unbiased counting rules have been considered the gold standard for estimation of the total number of TH+ neurons in a given brain structure (Eriksen et al., 2009; Gundersen et al., 1988; Schmitz and Hof, 2005; West et al., 1991). Although stereological approaches have provided the most consistent results on cell counts, the method is labor-intensive and time-consuming and therefore most often applied for quantitative assessment of TH+ neurons in discrete areas of the midbrain (Baquet et al., 2005; Chermenina et al., 2015; Fabricius et al., 2017; Garcia-Reitboeck et al., 2013; Hansen et al., 2016; Joyce et al., 2004; Komnig et al., 2016; Pakkenberg et al., 1995; Smeyne et al., 2016). Similar limitations apply to determination of dopaminergic projections estimated by manually counting visible TH+ fibers (Straub et al., 2008), which are therefore usually evaluated semi-quantitatively by 2D optical density analysis on thin sections (Joyce et al., 2004; Komnig et al., 2016). Hence, there is a need for methods enabling comprehensive and more efficient quantitative analysis of dopaminergic cell architecture changes in animal models of PD.

In recent years, there have been considerable advances in the development of methods that allow whole-organ immunolabeling (Liebmann et al., 2016; Renier et al., 2014, 2016). In combination with solvent-based tissue clearing methods and light-sheet fluorescence microscopy (LSFM) it is therefore possible to scan entire organs with cellular resolution (Becker et al., 2012; Epp et al., 2015; Susaki et al., 2015). Critical for the successful implementation of deep-tissue LSFM and 3D image reconstruction is optimal tissue clearing for complete visualization of cell topography. Among the various techniques available, immunolabeling-enabled three-dimensional imaging of solvent-cleared organs (iDISCO) has emerged as a simple and rapid method for achieving good antibody penetration and tissue clearing (Liebmann et al., 2016; Renier et al., 2014; Renier et al., 2016). iDISCO–LSFM is particularly useful for CNS research as the low brain anatomical variation between individual mice allows scanned brains to be mapped onto a common coordinated framework (CCF) for subsequent unbiased quantitative image analysis of immune-labeled molecular targets (Detrez et al., 2019; Hasegawa et al., 2019; Henning et al., 2019; Kjaergaard et al., 2019; Liebmann et al., 2016; Merz et al., 2019; Mzinza et al., 2018; Renier et al., 2016).

In the present study, we applied whole-organ immunolabeling and LSFM in combination with deep learning-assisted image analysis for fully automated registration and quantification of TH+ cells in the mouse brain. We subsequently applied this method for atlas-guided whole-brain quantification of dopaminergic neuronal pathways in the MPTP mouse model of PD.

## RESULTS

### A complete 3D map of TH-positive cells in the intact normal mouse brain

**Fig. 1.**
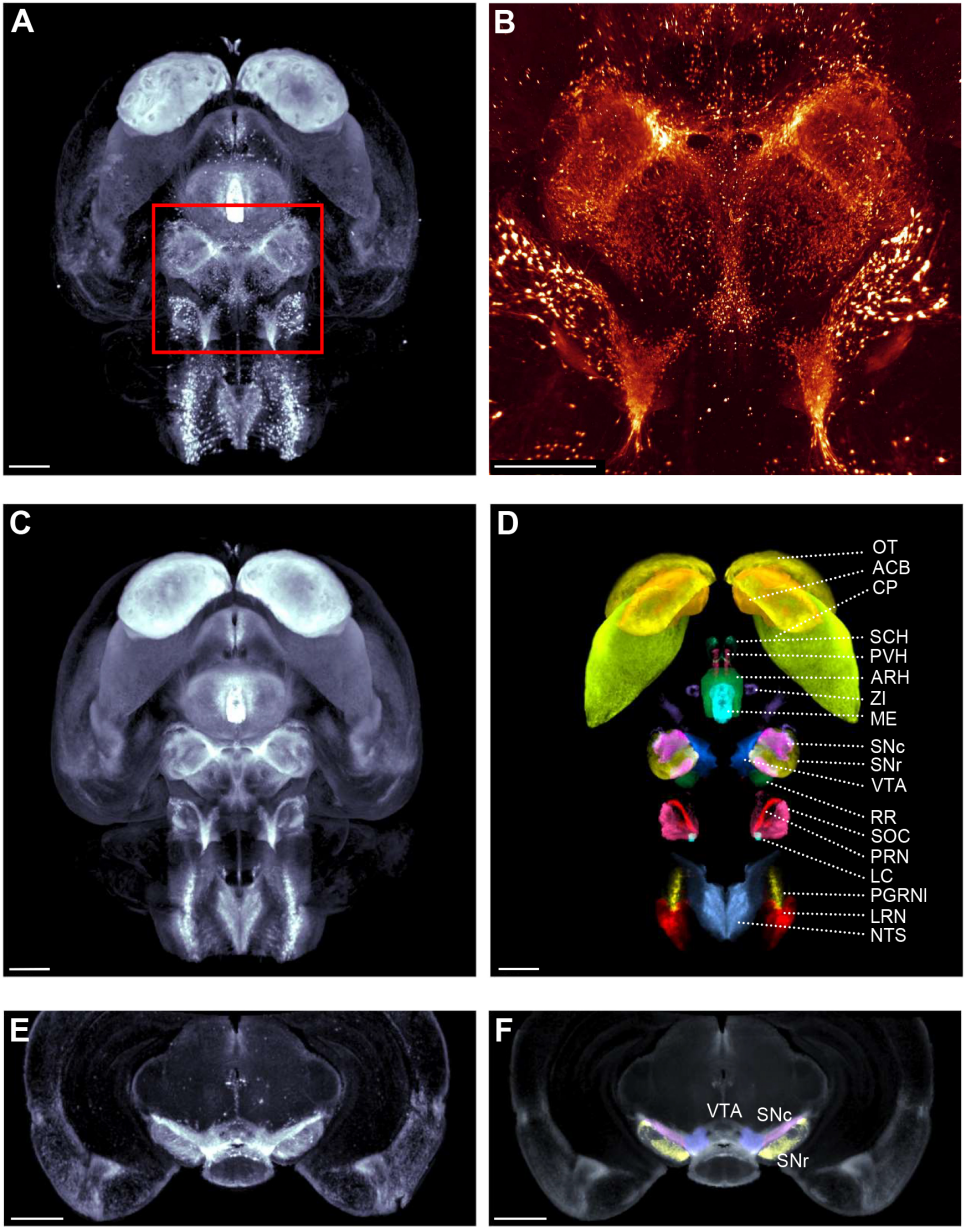
**Generation of brain-wide tyrosine hydroxylase expression map in the mouse.** (A) Light-sheet fluorescence brain imaging of tyrosine hydroxylase (TH) expression in representative vehicle-dosed control mouse. (B) Further magnification (5×) of boxed midbrain area in panel A. (C) Mean fluorescence intensity TH expression pattern, generated from seven individual mouse brains. (D) Map of average TH expression in major catecholaminergic brain regions (colour-coded for easier visualization). (E,F) Virtual cross-section through the midbrain region of the mean fluorescence intensity image of the mouse brain, depicting conspicuous TH expression in the VTA, SNc and SNr. ACB, nucleus accumbens; ARH, arcuate nucleus; CP, caudate–putamen; LRN, lateral reticular nucleus; LC, locus coeruleus; ME, median eminence; NTS, nucleus of the solitary tract; OT, olfactory tubercle; PGRNl, paragigantocellular nucleus; PRN, pontine reticular formation; PVH, paraventricular nucleus; RR, retrorubral field; SNc, substantia nigra pars compacta; SNr, substantia nigra pars reticulata; SCH, suprachiasmatic nucleus; SOC, superior olivary complex; VTA, ventral tegmental area; ZI, zona incerta. Scale bars: 1 mm.

We first generated a complete 3D map of TH+ neurons in vehicle-injected mice (*n*=7). We found that glyoxal fixation was optimal for preservation of whole-brain TH staining (Richter et al., 2018) and was highly compatible with iDISCO–LSFM imaging (Fig. 1A,B; Movies 1 and 2). For anatomical annotation of TH+ cells, each scanned mouse brain was mapped onto a CCF using the autofluorescence channel for anatomical registration. Once registered into the same spatial reference, an average mouse whole-brain 3D map of TH expression was generated (Fig. 1C). TH+ neurons were consistently detected in all major catecholaminergic cell groups (Fig. 1D). For clarity, both anatomical names and standard nomenclature of catecholaminergic cell groups (Dahlström and Fuxe, 1964; Hökfelt et al., 1984) were applied to describe these brain regions. Midbrain dopaminergic pathways were clearly delineated by TH staining. The SNc (A9) and ascending projections to the caudate–putamen (CP) showed dense TH-staining. Strong TH labeling was also associated with the mesolimbic pathway, including the ventral tegmental area (VTA, A10) and terminal projection areas in the nucleus accumbens (ACB) and olfactory tubercle. Midbrain TH+ neurons were also determined in the retrorubral field (A8). At the level of the hypothalamus, high TH signal intensity was associated with the tuberoinfundibular dopaminergic pathway, arising in the arcuate nucleus (ARH, A12) with dense projecting fibers in the median eminence (ME). A discrete population of TH+ cells was present in the zona incerta (A13), indicative of dopaminergic neurons in the incertohypothalamic pathway. In addition, the paraventricular nucleus (A11), hypothalamic preoptic area (A14–A15) and suprachiasmatic nucleus were positive for TH staining. TH staining was also clearly detected in noradrenergic nuclei of the brain stem, including the pontine reticular formation (PRN, A7), locus coeruleus (LC, A6), superior olivary complex (SOC, A5), nucleus of the solitary tract (NTS, A2) and lateral reticular nucleus (LRN, A1). Strong TH immunoreactivity was also observed in the lateral paragigantocellular nucleus (PGRNl). The principal catecholaminergic neurotransmitter phenotype in TH-expressing nuclei was confirmed by cross-referencing our TH imaging data (Fig. 1E,F) with *in situ* mRNA hybridization data imported from the Allen Mouse Brain Atlas (https://mouse.brain-map.org). These included transcriptional markers of dopaminergic (TH and dopamine transporter) and noradrenergic (dopamine beta-hydroxylase and noradrenaline transporter) neurons (Fig. S1).

### The MPTP mouse model of PD displays widespread changes in TH expression

**Fig. 2.**
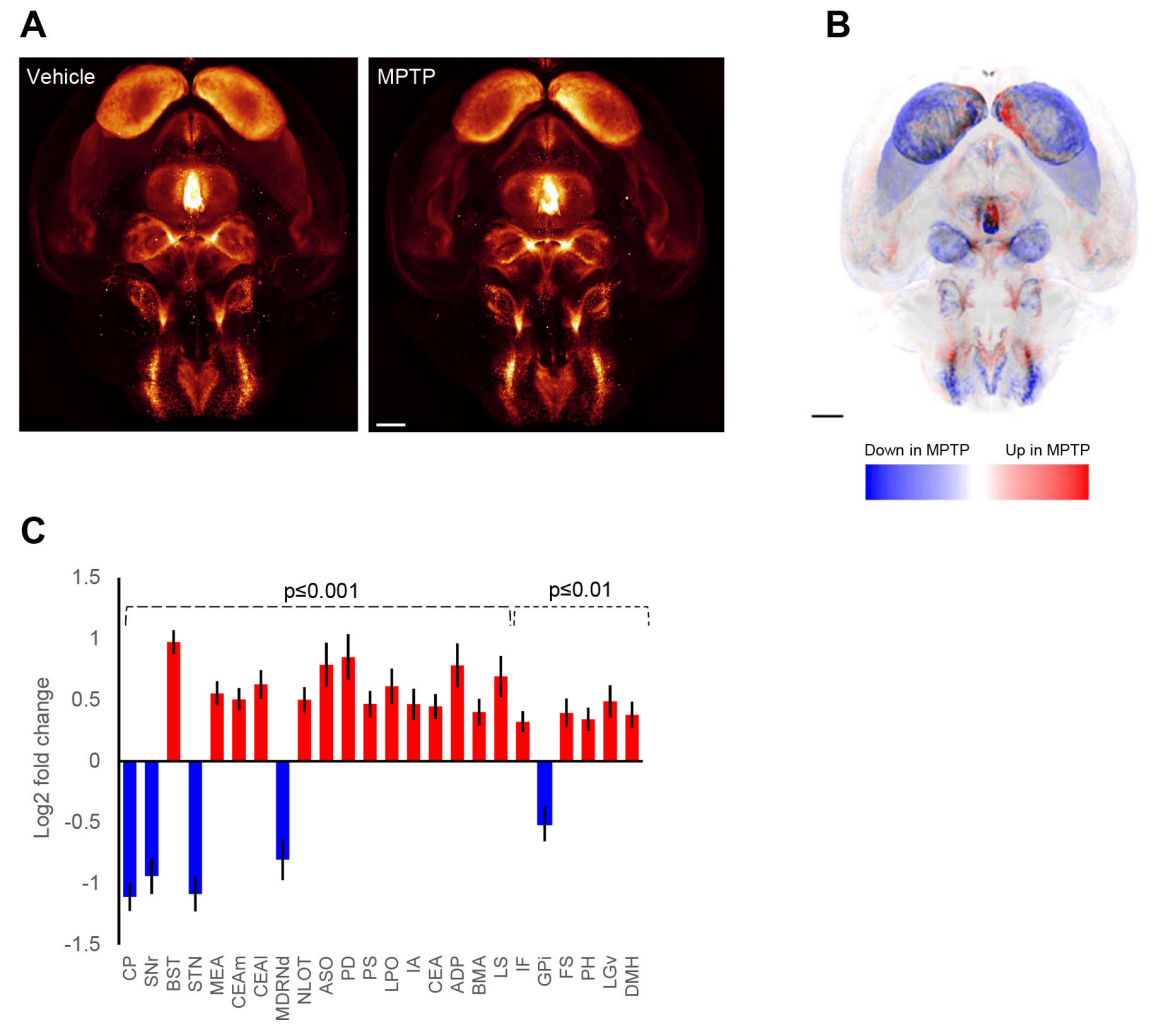
**Automated whole-brain imaging of MPTP-induced changes in tyrosine hydroxylase TH expression in the mouse.** (A) Mean fluorescence intensity signature of TH expression in vehicle control (left panel) and MPTP-dosed (right panel) mice. TH expression from each scanned brain was transferred to an average brain 3D coordinate mesh with mean expression calculated for each voxel. (B) Visualization of mean change in TH expression in MPTP-dosed mice (*n*=10) compared to vehicle-dosed control mice (*n*=7). Brain regions with altered average TH signal intensity are delineated in blue (downregulation) or red (upregulation). (C) Fold change (log_2_ scale) in TH expression for every brain region of MPTP-dosed mice compared to vehicle controls. Only regions that show a statistically significant change between the groups are indicated (*P*<0.01 and *P*<0.001; unpaired two-tailed *t*-test). False discovery rate correction was applied. Brain regions are ranked by statistical significance from left to right. ADP, anterodorsal preoptic nucleus; ASO, accessory supraoptic group; BMA, basomedial amygdala nucleus; BST, bed nucleus of stria terminalis; CP, caudate–putamen; CEA, central amygdala nucleus (i.e. both CEAl and CEAm); CEAl, central amygdala nucleus, lateral part; CEAm, central amygdala nucleus (medial part); FS, fundus of striatum; DMH, dorsomedial nucleus of the hypothalamus; GPi, globus pallidus (internal segment); IA, intercalated amygdala nucleus; IF, interfascicular nucleus raphe; LGv, lateral geniculate complex (ventral part); LPO, lateral preoptic area; LS, lateral septal nucleus; MDRNd, medullary reticular nucleus (dorsal part); MEA, medial amygdala nucleus; NLOT, nucleus of the lateral olfactory tract; PD, posterodorsal preoptic nucleus; PH, posterior hypothalamic nucleus; PS, parastrial nucleus; SNr, substantia nigra pars reticulata; STN, subthalamic nucleus. Scale bars: 1 mm.

**Fig. 3.**
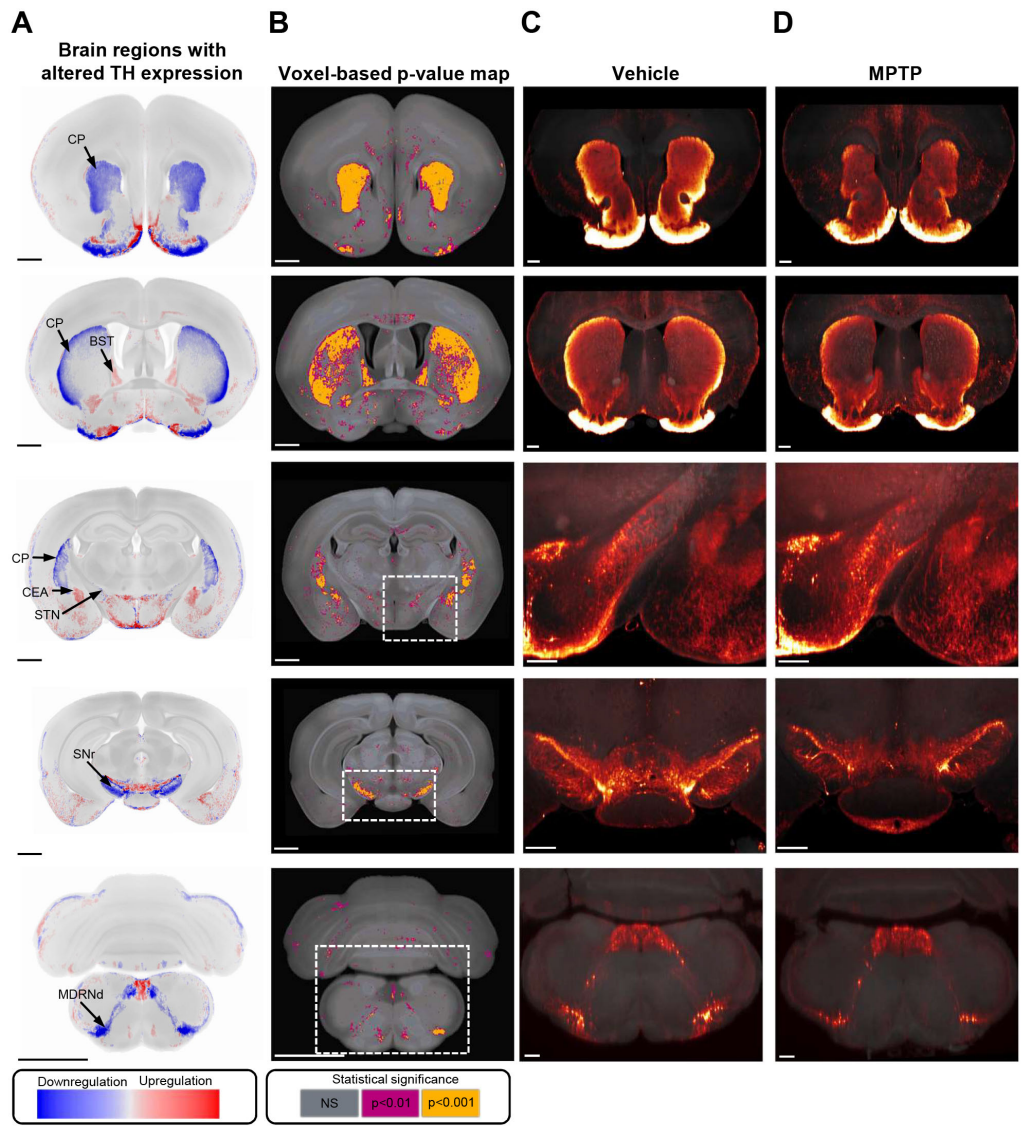
**Automated voxel-based whole-brain quantitative analysis of changes in tyrosine hydroxylase expression in MPTP-dosed mice.** (A) Virtual coronal sections (20 µm) from 3D reconstructed average MPTP mouse whole brain (see also Fig. 2B). MPTP-dosed mouse brain regions with significantly altered mean TH signal intensity are delineated in blue (downregulation) or red (upregulation). Virtual coronal sections sampled at the level of forebrain, midbrain and brainstem are shown. (B) Voxel-based statistical analysis performed on 3D-imaged brains. Brain regions in MPTP-dosed mice with significant regulation of TH expression are indicated, as compared to vehicle controls (*P*<0.01 and *P*<0.001; unpaired two-tailed *t*-test; NS, not significant). (C) Representative light-sheet fluorescence images from vehicle control mouse. (D) Representative light-sheet fluorescence images from MPTP-dosed mouse. CEA, central amygdala nucleus; CP, caudate-putamen; BST, bed nucleus of stria terminalis; MDRNd, medullary reticular nucleus (dorsal part); STN, subthalamic nucleus; SNr, substantia nigra pars reticulata. Scale bars: 1 mm in A,B; 500 µm in C,D.

The initial whole-brain 3D mapping of TH+ catecholaminergic neurons in normal control mice served as a basis for in-depth characterization of the TH+ cell architecture in the MPTP mouse model of PD. MPTP-dosed mouse brains (*n*=10) were stained for TH, scanned and mapped into the same CCF as the control group. Overview of TH staining in all individual brains from vehicle- and MPTP-dosed mice is shown in Fig. S2. Because all mouse brains were mapped to the same spatial framework, group-wise differential TH signal intensity can be illustrated by subtracting mean brain regional signal intensities from vehicle controls and MPTP-dosed mice, respectively (Fig. 2A,B; Movies 3 and 4). The statistical analysis compared TH signal intensities in all individual mouse brains (vehicle, *n*=7; MPTP, *n*=10) and included 276 individual brain regions. Of these, a total of 24 regions showed significant changes in TH signal intensity following acute MPTP administration (Fig. 2B,C). To provide an overview of the brain-wide changes in TH expression in MPTP-dosed mice, brain regions with significantly altered mean TH signal intensity (*P*<0.01 vs vehicle control) are delineated in blue (downregulation) or red (upregulation), see Fig. 2B,C and Fig. 3A. Because direct voxel-based visualization of changes in TH signal intensity may inaccurately estimate brain regional differences in vehicle vs MPTP-dosed mice, we generated a voxel-based *P*-value map to only consider statistically significant changes (Fig. 3B). Representative images from individual mice are shown in Fig. 3C,D. In MPTP-treated mice, significantly reduced TH signal intensity was almost exclusively detected in major regions of the basal ganglia, including the CP, substantia nigra pars reticulata (SNr), subthalamic nucleus (STN) and internal segment of globus pallidus (GPi). In addition, the medullary reticular nucleus (dorsal part, MDRNd) showed significantly reduced TH expression. In contrast, increased TH signal intensity in MPTP-dosed mice was predominantly observed in various limbic structures, including several subdivisions of the amygdala [central, medial and/or basomedial nuclei (CEA, MEA, BMA), intercalated amygdala nucleus (IA) and hypothalamus (anterodorsal and/or posterodorsal preoptic nucleus, lateral preoptic area, accessory supraoptic group, posterior hypothalamic nucleus, dorsomedial nucleus of the hypothalamus]. TH signal intensity was unchanged in all other areas of the hypothalamus, including nuclei representing the tuberoinfundibular dopaminergic pathway (ARH and ME). Other limbic regions, i.e. the lateral septal nucleus (LS) and bed nucleus of the stria terminals (BST), also showed significantly upregulated TH signal intensity. In addition, MPTP-treated mice showed significantly increased TH signal intensity in discrete areas of the thalamus (ventral part of the lateral geniculate complex, LGv) and brain stem (interfascicular nucleus raphe, IF).

### Unbiased counting of TH-positive cells

**Fig. 4.**
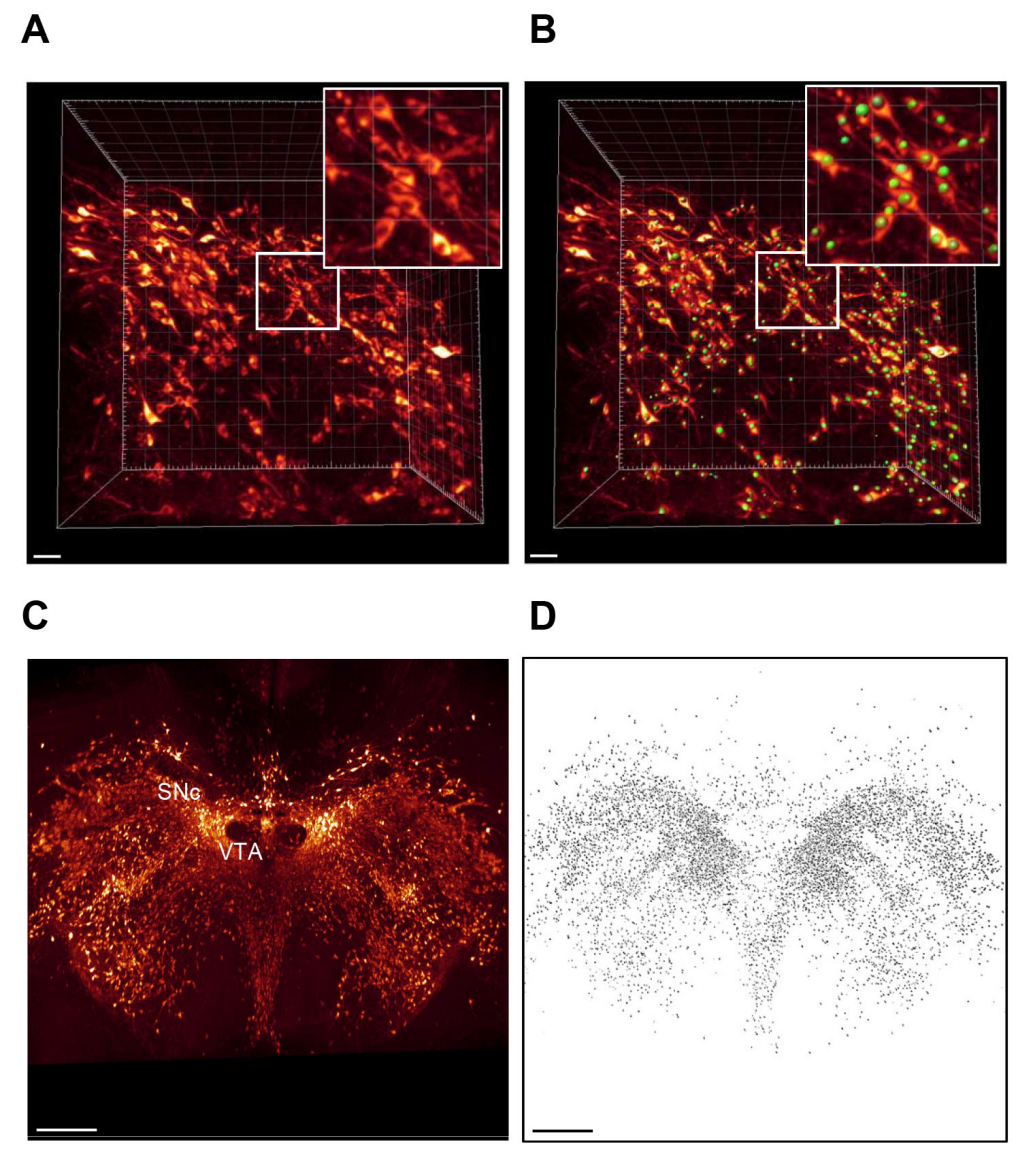
**Development of deep learning-based method for counting of tyrosine hydroxylase-positive cells in the mouse midbrain.** (A) Cell nuclei distinguishable in high-resolution scan of TH-stained mouse brain. (B) Deep-learning computational model applied to high-resolution midbrain scans for automated identification and registration of nuclei in TH-expressing cells. Boxed area is magnified in upper right corner. (C,D) Automated detection of TH-positive cells in midbrain whole 3D-image stack from vehicle-dosed mouse. Panel C, dorsal view; panel D, corresponding map of TH-positive cells detected in the sample. SNc, substantia nigra pars compacta; VTA, ventral tegmental area. Scale bars: 500 µm.

**Fig. 5.**
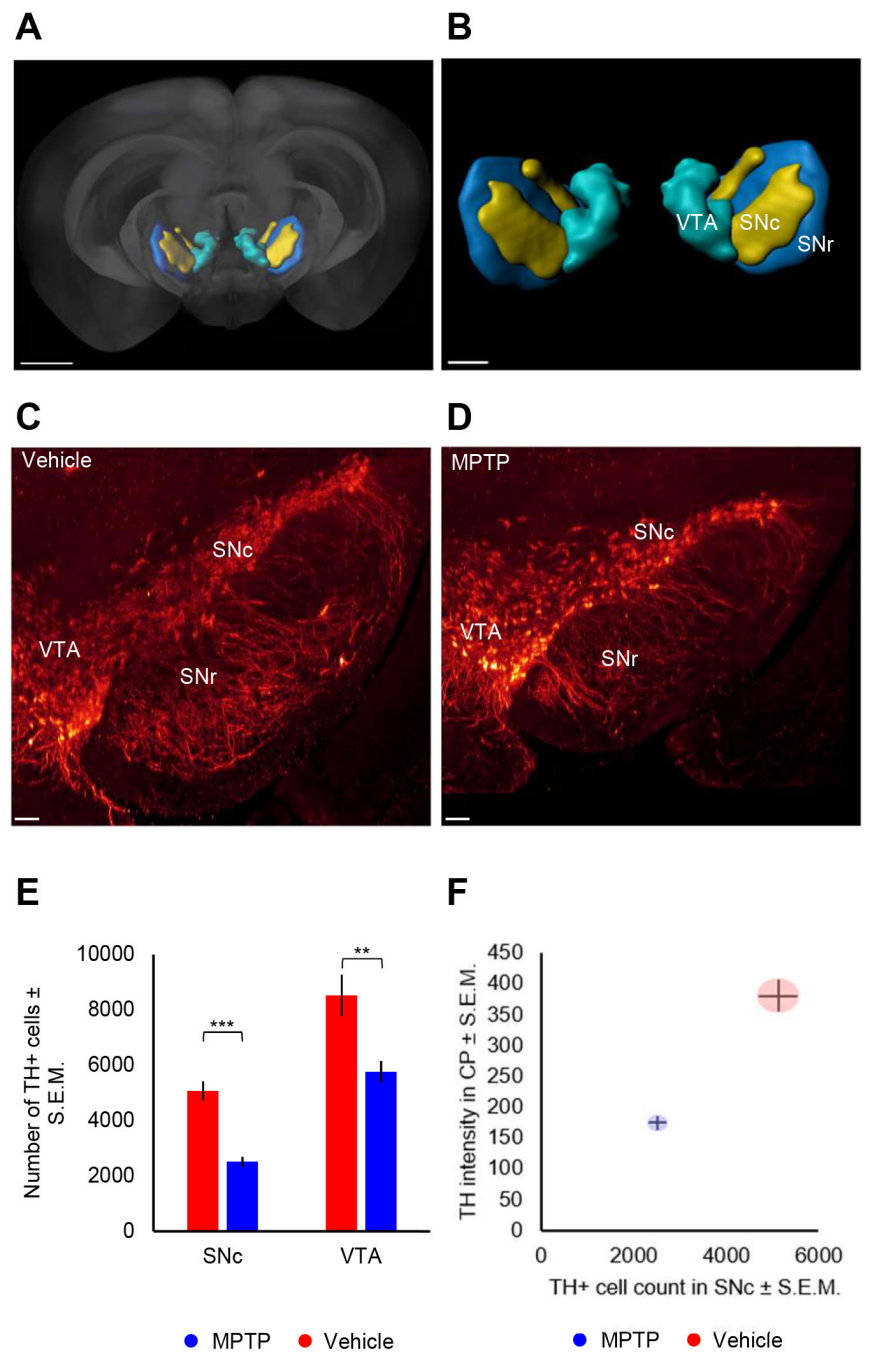
**3D cell quantification of tyrosine hydroxylase-positive cells in the mouse midbrain.** (A,B) Visualization of 3D coordinates of midbrain dopaminergic areas in mouse brain. False-colored region in A is magnified in B. (C,D) Coronal midbrain sections constructed from the whole 3D-image stack in representative vehicle (C)- and MPTP (D)-dosed mice, respectively. (E) Automated deep learning-based counting of TH-positive neurons in the SNc and VTA. **P<0.01; ***P<0.001 (one-way ANOVA, compared to vehicle controls). (F) TH signal intensity in the caudate–putamen plotted against corresponding number of TH-positive cells in the SNc. CP, caudate–putamen; SNc, substantia nigra pars compacta; SNr, substantia nigra pars reticulata; VTA, ventral tegmental area. Scale bars: 100 µm.

Because unbiased estimation of TH+ dopaminergic neuron numbers is crucial for phenotyping and histological assessment of treatment effects in PD models, we sought to extend the iDISCO–LSFM imaging method to permit automated counting of TH+ neurons in specific mouse midbrain areas. To facilitate this, the midbrain region was re-scanned at higher magnification to delineate and count individual TH+ cells in the SNc and VTA (Movies 5 and 6). This enabled identification of single TH+ neurons and the corresponding cell nucleus (appearing as a black sphere surrounded by cytoplasmic TH-staining), see Fig. 4A. This particular feature was used to generate and train a U-net based deep learning algorithm to consistently identify TH+ neurons in the SNc and VTA (Fig. 4B). The algorithm was applied to high-resolution midbrain scans from vehicle (*n*=7)- and MPTP (*n*=9)-dosed mice (Fig. 4C,D). In order to perform atlas-guided assignment of TH+ neurons each individual high-magnification midbrain scan was mapped back into the corresponding low-resolution brain scan containing the atlas rendering of SNc and VTA (Fig. 5A,B). Using this approach, a total of 5073±346 (mean±s.e.m.) and 8526±750 TH+ cells were determined in the SNc and VTA, respectively, in vehicle-dosed mice. Following MPTP dosing, the number of TH+ cells was significantly reduced to 2517±190 (SNc, *P*<0.001) and 5761±417 (VTA, *P*<0.01) (Fig. 5E). This effect was also evident in coronal sections constructed from the whole 3D image stack, demonstrating less dense TH+ cell populations in these two dopaminergic midbrain regions (Fig. 5C,D). In general, MPTP-induced loss of TH+ neurons appeared most pronounced in the lateral SNc (Fig. 5C,D). The loss of TH+ SNc cells in MPTP-dosed mice closely correlated to reduced TH signal intensity in the CP (Fig. 5F), the major target region for dopaminergic SNc neurons.

### Impaired motor coordination as a predictor of TH+ neuronal loss in the substantia nigra

**Fig. 6.**
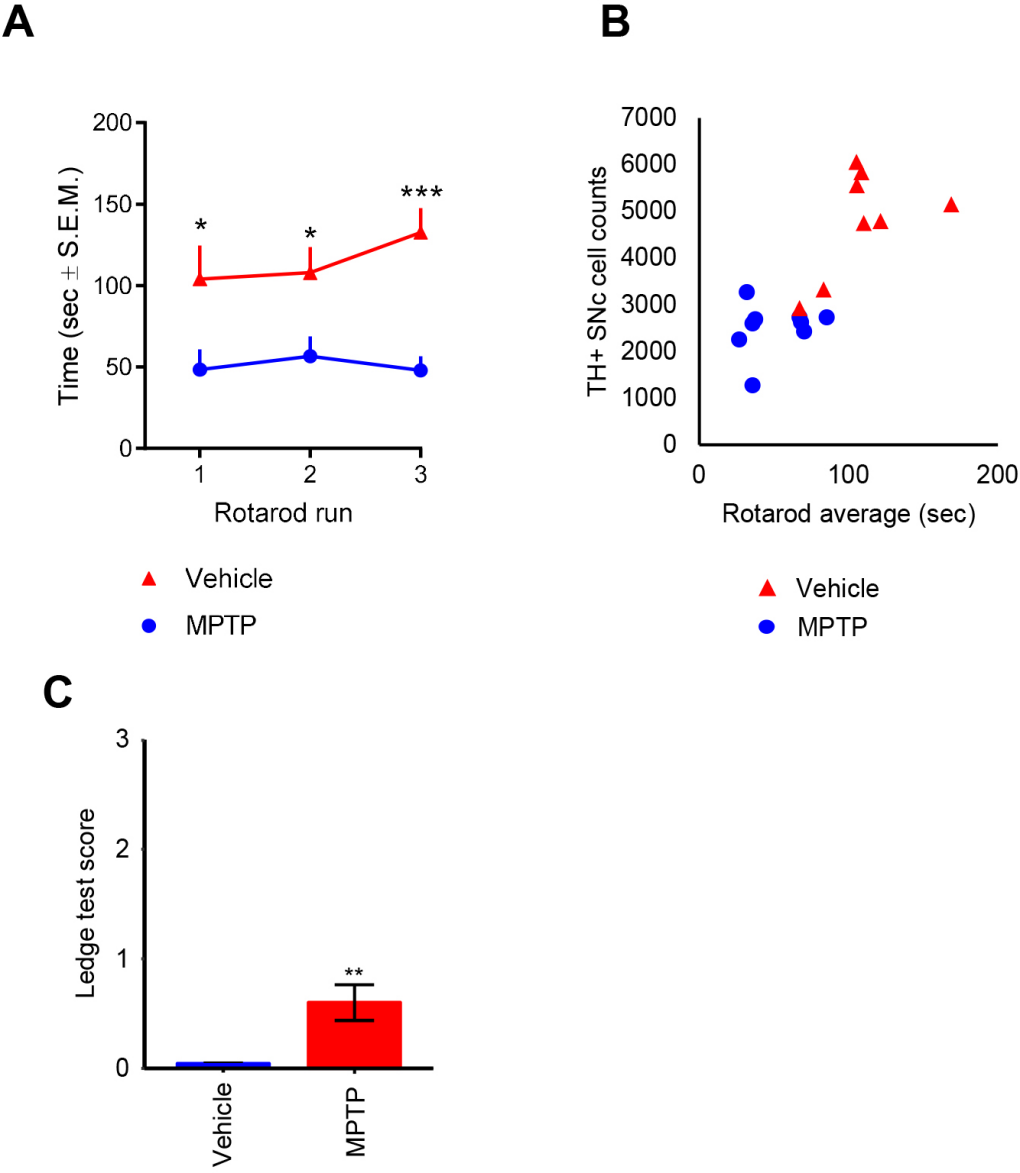
**Correlation** **between** **tyrosine hydroxylase-positive cells and motor coordination skills.** (A) Time spent on rotarod over three consecutive tests separated by 30 min (performed on study day 6). *P<0.05; ***P<0.001 (Dunnett's test on two-factor linear regression model with interaction, compared to vehicle controls). (B) Number of TH-positive cells in the substantia nigra pars compacta (SNc) plotted against mean rotarod test performance in vehicle (*n*=7)- and MPTP (*n*=9)-dosed mice (*r*=0.77). Impaired rotarod performance was observed at ≥50% depletion of TH-positive SNc cells compared to mean level in vehicle control mice. (C) MPTP-dosed mice show impaired performance compared to vehicle-dosed mice in a composite motor behavioral test. **P<0.01 (unpaired *t*-test).

Motor coordination was assessed in a rotarod test on study day 6 (the day before termination) in MPTP-dosed mice (*n*=9) and vehicle controls (*n*=7). Compared to vehicle controls, MPTP-dosed mice demonstrated significantly impaired rotarod performance in all three successive tests (Fig. 6A). There was a strong positive correlation between SNc cell counts and mean rotarod performance (*r*=0.79) (Fig. 6B). Also, MPTP-dosed mice showed significant, albeit mild, motor performance deficits in a composite behavioral phenotyping test (*P*<0.01) (Fig. 6C).

## DISCUSSION

Here, we present the first quantitative LSFM 3D imaging study on TH expression in the intact adult mouse brain. When combined with deep learning-based computational image analysis, this pipeline enabled detection of comprehensive quantitative changes in catecholaminergic pathways in the MPTP mouse model of PD. Additionally, the platform facilitated unbiased, automated and rapid counting of TH-positive neurons in dopaminergic midbrain regions of the MPTP mouse. Our study therefore sets a framework for using high-throughput whole-brain 3D imaging in preclinical drug discovery for PD and other dopaminergic disease states.

Using LSFM and computational quantification of the specific fluorescence signal from several mouse brains, we first established the average TH expression pattern. Notably, LSFM enabled whole-brain visualization of the complex architecture of the nigrostriatal and mesolimbic dopaminergic pathways, originating in the substantia nigra–VTA complex with dense ascending projections innervating several motor and limbic structures. Consistent with dopaminergic neurons being a key integrative component in basal ganglia circuits (Rice et al., 2011; Smith and Kieval, 2000), TH expression was clearly detected in the SNc, CP, GP and STN. Although the GP and STN are not considered among the principal nigrostriatal dopaminergic projection pathways, TH expression has been reported previously in discrete areas of the GP and STN (Hassani et al., 1997; He et al., 2016; Lindgren et al., 2012; Lindvall and Björklund, 1979). As the GP has been reported to be traversed by nigrostriatal axon bundles (Lindvall and Björklund, 1974), TH-expressing axonal projections not terminating in the GP may potentially have contributed significantly to the TH signal in this region.

TH staining was also observed in the arcuate nucleus and zona incerta, representing key nuclei of the hypothalamic tuberoinfundibular and incertohypothalamic dopaminergic pathways regulating prolactin secretion (Grattan, 2015) and locomotor activity (Fougère et al., 2019), respectively. Our results are in agreement with previous reports on TH expression determined by conventional 2D immunohistochemical (Araki et al., 2001; Bucci et al., 2017; D'Este et al., 2007; Jaber et al., 1999; Nelson et al., 1997; Ruggiero et al., 1984) and *in situ* mRNA hybridization techniques (Baker et al., 2003; Choi et al., 2012; Huang et al., 2005; Jakowec et al., 2004; Weiss-Wunder and Chesselet, 1991) (see Fig. S1). In addition, the topography of dopaminergic pathways visualized by 3D mapping of TH expression in the adult mouse brain corresponds to previous iDISCO–LSFM qualitative studies on central TH distribution in the embryonic, neonatal and adult mouse (Godefroy et al., 2017, 2019; Renier et al., 2014). Also consistent with recent iDISCO–LSFM imaging analysis of the neonatal mouse brain (Godefroy et al., 2017, 2019), significant TH expression was detected in distinct regions of the adult mouse brain stem, specifically in the PRN, LC, SOC, NTS, LRN and PGRNl. These sympathoexcitatory areas are densely populated by noradrenaline- and adrenaline-producing neurons involved in behavioral modulation and central autonomic control (Berridge and Waterhouse, 2003; Guyenet, 1991; Szabadi, 2013). To provide further information on molecular signaling, our 3D whole-brain reference map of TH expression can be superimposed with other molecular targets, e.g. cell activation markers (c-Fos), determined by iDISCO–LSFM (Salinas et al., 2018).

Dopamine neurons have been intensely studied in humans and animal models of neurodegenerative diseases, as deficient function of dopaminergic neuron clusters in the SNc–VTA complex is closely related to the pathogenesis of PD and plays an important role in a range of other CNS diseases such as Huntington's disease and schizophrenia, as well as food and drug addiction (Grace and Gomes, 2019; Kenny, 2011; Klein et al., 2019; Obeso et al., 2008; Volkow et al., 2017). Unbiased estimates of TH+ dopaminergic neuron numbers is fundamental in the assessment of histopathological changes and neuroprotective drug treatment effects in preclinical models of PD (Fabricius et al., 2017). Previous LSFM studies have only provided a qualitative view on the distribution of TH-expressing neurons in individual mouse brains (Godefroy et al., 2017, 2019; Renier et al., 2014). To perform whole-brain quantitative analyses of TH expression, we established a deep learning-based LSFM platform for automated counting of TH+ neurons. Using U-net for image segmentation (Ronneberger et al., 2015), we developed and trained a cell-detection algorithm for automated registration of midbrain TH+ neurons with a visible nucleus. Considering the dense population of midbrain dopaminergic cell bodies, the algorithm was optimized to enable automated bilateral counting of TH+ perikarya in the SNc and VTA at cellular resolution. Accordingly, vehicle-dosed control mice displayed a total number of TH+ cells in the SNc and VTA, being well within the range of total TH+ cell counts reported in C57BL/6J mice using stereological methods (Baquet et al., 2009; Benskey et al., 2013; Bucci et al., 2017; Chermenina et al., 2015; Garcia-Reitboeck et al., 2013; Jakowec et al., 2004; Joyce et al., 2004; Liang et al., 2019; Smeyne et al., 2016). Compared to vehicle controls, MPTP dosing led to significant depletion of TH+ neurons in both the SNc (50% reduction) and VTA (33% reduction). Reduced striatal TH+ fiber density has also been reported previously in MPTP-dosed mice (Dong et al., 2002; Hayley et al., 2004; Joyce et al., 2003; Komnig et al., 2016; von Bohlen und Halbach et al., 2005).

In MPTP-dosed mice, the lowered level of TH staining intensity in the CP closely corresponded with loss of TH+ cells in the SNc, which most likely signifies loss of TH+ CP fibers originating in the SNc. Furthermore, MPTP-induced motor deficits in the rotarod test were observed at ≥50% depletion of TH+ cells in SNc. This implies that impaired rotarod performance could provide an index with relatively good predictive value of nigral lesion progression in MPTP-dosed mice, and further supports the notion that impaired motor coordination in MPTP-dosed mice is due to deficient nigrostriatal connectivity (Rozas et al., 1998).

Characterization of preclinical animal models of neurodegenerative disorders has typically focused on histological and molecular analyses in predefined brain regions, which may lead to incomplete understanding of the complex neuropathology in preclinical neurodegenerative disease models. As we demonstrate here, automated quantitative whole-brain volumetric analysis tracked widespread MPTP-induced changes in TH expression. Reduced TH signal intensity was largely confined to basal ganglia nuclei, including SNr, CP and STN. In contrast to the marked MPTP-induced loss of SNc TH+ cells, we did not detect significantly altered SNc TH intensity levels in MPTP-dosed mice, suggesting compensatory upregulation of TH expression in spared TH+ SNc neurons. Also, the different parameters and sensitivity of the two methodologies should be considered. Whereas TH+ cell counting specifically addresses histomorphological changes, the quantitative volumetric analysis captures mean TH signal intensity (expression) at lower resolution. As TH+ neuronal loss was most pronounced in the lateral SNc, quantitative volumetric analysis applied to more discrete areas of the SNc may therefore prove to be more sensitive.

Interestingly, increased TH signal intensity was largely confined to limbic brain regions, including several nuclear subdivisions of the amygdala and hypothalamus. The amygdala receives dopaminergic inputs from the SNc and VTA, and abnormal amygdala activity has been implicated in emotional deficits in PD (Ibarretxe-Bilbao et al., 2009; Tessitore et al., 2002). Our data in 10-week-old MPTP-dosed mice contrasts with a previous report on reduced TH+ fiber density in the amygdala following acute MPTP administration in 12-month-old mice (von Bohlen und Halbach et al., 2005). Considering that susceptibility to MPTP toxicity increases with age in C57BL6/J mice (Filipov et al., 2009; Irwin et al., 1993), it is possible that only young MPTP-dosed mice display upregulation of TH signal intensity in the amygdala. Our LSFM quantitative analysis also revealed that TH expression was upregulated in a subset of dorsal and posterior nuclei of the hypothalamus following MPTP administration. Correspondingly, increased hypothalamic adrenaline levels have been reported in MPTP-dosed mice (Sunström et al., 1987). In contrast, TH signal intensity in the mediobasal hypothalamus, including the ARH and ME (which make up the intrinsic tuberoinfundibular dopaminergic pathway) was unchanged in MPTP-dosed mice. Consistent with tuberoinfundibular dopaminergic neurons remaining unaffected in individuals with PD (Braak et al., 2003; Langston and Forno, 1978), the ME in MPTP-dosed mice shows rapid reversal (within 24 h) of TH and dopamine loss (Benskey et al., 2012; Benskey et al., 2013). Dopamine levels in the paraventricular nucleus also show rapid recovery (within 12 h) after MPTP administration (Di et al., 2019). Our finding of sustained upregulation of TH signal intensity in several other hypothalamic areas points to spatiotemporal differences in hypothalamic TH expression in MPTP-dosed mice, and further emphasizes that regulation of central TH expression in this standard mouse model of PD is more complex than anticipated from previous conventional histological studies.

Our quantitative 3D imaging study in MPTP-dosed mice revealed subtle, yet statistically significant, upregulation of TH signal intensity in several limbic brain regions, which emphasizes the robustness and sensitivity of the LSFM–deep learning pipeline. Several of these brain areas have not previously been implicated in MPTP-induced toxicity and the potential functional implications must therefore await future studies. Nevertheless, increased understanding of different compensatory molecular mechanisms in PD is considered an important strategy for promoting new treatments (Obeso et al., 2004). Notably, spatiotemporal changes in dopamine concentrations and receptor expression are mechanisms presumed to delay the onset of cardinal motor symptoms of PD (Arkadir et al., 2014; Gerfen, 2003). In line with this notion, increased TH expression has been observed in discrete areas of the VTA in individuals with PD (Tong et al., 2000), and studies in MPTP non-human primate models have demonstrated that increased striatal TH expression peaks before the manifestation of motor symptoms in PD (Bubak et al., 2015; Tandé et al., 2006). Although TH-associated adaptive changes in the limbic system have not been described in PD, neuronal plasticity in the hypothalamus has been proposed to play a role in the neuroendocrine disturbances in PD (Ansorge et al., 1997; Caudal et al., 2015). We therefore speculate that MPTP-induced upregulation of TH signal intensity in limbic projection areas could represent an additional molecular adaptive mechanism compensating for progressive loss of dopamine-producing neurons in the midbrain.

In conclusion, our combined LSFM–deep learning platform permitted fully automated 3D mapping and unbiased quantification of TH+ cells throughout the intact adult mouse brain. The pipeline revealed widespread and complex changes in central catecholaminergic pathways in the MPTP mouse model of PD. This approach may therefore be applied for characterizing brain-wide effects of compounds targeting dopaminergic neurons in mouse models of PD, as well as in other mouse models of CNS diseases with deficient dopaminergic neurotransmission.

## MATERIALS AND METHODS

### Animals

The Danish Animal Experiments Inspectorate approved all experiments, which were conducted using internationally accepted principles for the use of laboratory animals (license #2013-15-2934-00784). Male C57BL/6JRj mice (10 weeks old) were from Janvier Labs (Le Genest Saint Isle, France) and group-housed (five mice per cage) in a controlled environment (12 h light:dark cycle, lights on at 03:00, 21±2°C, humidity 50±10%). Each animal was identified by an implantable subcutaneous microchip (PetID Microchip, E-vet, Haderslev, Denmark). Mice had *ad libitum* access to tap water and chow (Altromin 1324, Brogaarden, Hoersholm, Denmark). Mice received four intraperitoneal injections of saline vehicle (*n*=7) or MPTP hydrochloride 20 mg/kg (*n*=10, Sigma-Aldrich, Soeborg, Denmark) administered as free base at 2 h intervals in a single day (day 0).

### Rotarod test

Mice were tested for cataleptic behavior 24 and 48 h after the last MPTP dose. The mouse was positioned on a vertical grid. Normal limb motor responsiveness was defined as no latency to correct the imposed posture. Motor coordination skills were evaluated using an accelerating rotarod (Panlab, Harvard Apparatus, Holliston, MA, USA). Four days prior to MPTP dosing, mice were trained on the rotarod and the latency to fall was measured in each of three successive tests (4–40 rpm over 5 min, tests separated by 30 min). The rotarod test was repeated six days post-MPTP dosing and expressed as mean latency to fall in each 5 min test period. The test was performed and recorded by an experimenter blind to treatment. Prior to rotarod testing on day 6, motor coordination and ataxia was scored using a composite phenotyping test (Guyenet et al., 2010), including a cage ledge test (score 0, normal; score 1, loses footing; score 2, poor use of hindlimbs; score 3, falls off ledge), hindlimb clasping response during tail elevation (score 0, normal extension of both hindlimbs; score 1, one hindlimb retracted towards the abdomen; score 2, both hindlimbs partially retracted towards the abdomen; score 3, hindlimbs entirely retracted and touching the abdomen), and gait on a flat surface (score 0, body weight supported on all limbs and normal walking behavior; score 1, mild tremor or limp while walking; score 2, severe tremor, severe limp, lowered pelvis, or feet pointing away from the body during locomotion). All behavioral tests were performed and recorded by an experimenter blind to treatment.

### Tissue clearing and fluorescence labeling

Mice were anesthetized using Hypnorm-Dormicum (fentanyl 788 µg/kg, fluanisone 25 mg/kg and midazolam 12.5 mg/kg, s.c.), perfused intracardially with heparinized (15,000 IU/l) PBS for 2–3 min and cold (4°C) glyoxal fixative [20% ethanol, 8% glyoxal (128465, Sigma-Aldrich, Soeborg, Denmark) and 0.75% acetic acid in distilled water, pH 5.0]. The iDISCO procedure was essentially as previously reported (Renier et al., 2014) with slight modifications. Brains were carefully dissected and post-fixed in glyoxal fixative overnight at 4°C, thereafter equilibrated to room temperature for 1 h and washed in phosphate-buffered saline (PBS, pH 7.4) with shaking for 3×30 min at room temperature. Samples were dehydrated in a methanol–H_2_O gradient (20–40%, 60–80%, 100% methanol; each step 1 h at room temperature), washed in 100% methanol for 1 h and incubated overnight in 66% dichloromethane (DCM), 33% methanol at room temperature. Samples were then washed twice in 100% methanol for 30 min, cooled down to 4°C in 1 h and bleached in chilled fresh 5% H_2_O_2_ in methanol (1 volume 35% H_2_O_2_ to 6 volumes methanol) overnight at 4°C, then rehydrated in a methanol–PBS series (80%, 60%, 40%, 20% methanol, with 0.2% Triton X-100, 1 h each at room temperature). Samples were washed in PBS with 0.2% Triton X-100 (permeabilization solution) for 2×1 h at room temperature, incubated in permeabilization solution at 37°C for 3 days and blocking solution (0.2% Triton X-100, 6% donkey serum, 10% DMSO and 0.02% sodium azide in PBS) at 37°C for 2 days. Thereafter, samples were incubated with anti-TH antibody (1:500; AB152, Merck Millipore, Darmstadt, Germany) in antibody buffer (2% Tween-20, 5% DMSO, 3% donkey serum, 0.01 mg heparin/ml in PBS) at 37°C for 7 days followed by a series of washes in washing solution (2% Tween-20 and 0.01 mg heparin/ml in PBS; 10 min, 20 min, 30 min, 1 h, 2 h and 2 days). Samples were incubated with secondary antibody anti-rabbit-Cy5 (1:500; 711-175-152, Jackson ImmunoResearch, West Grove, PA, USA) in antibody buffer at 37°C for 7 days, then a series of washes in washing solution (10 min, 20 min, 30 min, 1 h, 2 h and 3 days). Samples were dehydrated in a methanol–H_2_O series (20%, 40%, 60%, 80%, 100% methanol) for 1 h at room temperature, then incubated in 100% methanol overnight in 66% DCM, 33% methanol for 3 h at room temperature followed by 100% DCM for 2×15 min twice (with shaking) to remove traces of methanol. The samples were finally transferred to dibenzyl ether (DBE) and stored in closed glass vials in the dark.

### Light-sheet microscopy

Brains were imaged using a Lavision light-sheet ultramicroscope II (Miltenyi Biotec GmbH, Bergisch Gladbach, Germany) with Zyla 4.2P-CL10 sCMOS camera (Andor Technology, Belfast, UK), SuperK EXTREME supercontinuum white-light laser EXR-15 (NKT Photonics, Birkerød, Denmark) and MV PLAPO 2XC (Olympus, Tokyo, Japan) objective. The brain was attached to a custom-made sample holder with neutral silicone (ventral side up) and imaged in a chamber filled with DBE. ImSpector microscope controller software (v7) was used (Miltenyi Biotec GmbH, Bergisch Gladbach, Germany). Horizontal images were acquired at 0.63× magnification (1.2× total magnification) with an exposure time of 228 ms in a *z*-stack at 7 µm intervals. Horizontal focusing was captured in seven planes with blending mode set to the center of the image to merge the individual raw images. Autofluorescence images were captured at 560±20 nm (excitation) and 650±25 nm (emission) wavelength (80% laser power in ImSpector software, 100% NKT laser). TH staining was imaged at 630±15 nm excitation wavelength and 680±15 nm emission wavelength (70% laser power in software, 100% mechanical). In addition, the midbrain area was scanned at 5× total magnification in two separate image tiles that were captured with 20% overlap and merged for subsequent analysis. To minimize experimental variation, all brains were processed using in-house standardized procedures. All brains were immunostained simultaneously using the same batches of primary and secondary antibodies, identical mounting position and orientation in the microscope, and similar scanning settings. Light sheets were aligned and calibrated once daily and alternating scanning order between vehicle controls and MTPT mice was applied.

### Image analysis

Region delineation of the whole-brain samples were obtained by atlas segmentation. Atlas annotations were obtained by alignment with the CCFv3 mouse brain atlas (Oh et al., 2014). The autofluorescence images from the TH-stained brains were down-sampled to 20 µm isotropic resolution and image contrast was enhanced by applying contrast limited adaptive histogram equalization (CLAHE). Registration between atlas and samples was performed as global affine alignment followed by local multi-resolution b-spline-based alignment. Elastix software (Klein et al., 2010) was applied to implement the registrations. Following manual estimation of the translation required, brain region segmentation of high-magnification midbrain scans (5×) was obtained by rigid registration between the high magnification sample and the corresponding full-brain scan. Registration was performed at 7 µm isotropic resolution. Spectral unmixing was performed to minimize the contribution of tissue autofluorescence in the acquired specific TH channel of the full brain samples. The estimated autofluorescence contribution in the specific channel was calculated and removed based on ratios of voxel intensities between selected voxels in the non-specific channel and the corresponding voxels in the specific channel. The unmix algorithm was applied in 10 µm isotropic resolution. An average TH signal density map was constructed for the vehicle (*n*=7)- and MPTP (*n*=10)-dosed groups by creating a voxel-wise mean average for each individual mouse in the groups aligned to the LSFM brain atlas space. Deep learning was used to locate TH+ neurons in the specific channel of the high-magnification midbrain scans from vehicle control mice (*n*=7) and MPTP-treated mice (*n*=9). U-net network architecture (Ronneberger et al., 2015) was used to create a 2D U-Net with four repeated layers for encoding and four repeated layers for decoding, implemented in Python utilizing the Keras machine learning library (https://github.com/keras-team/keras). The U-Net input was a single intensity channel and the output was a single label image. Annotations was performed manually on a total of 481 image tiles with a size of 320×320 pixels. 70% of the data were used for training, 20% for validation and 10% for testing. Data augmentation, in the form of skews, rotations, flips, zoom and random distortions was applied during training with increasing probability from 10–30% for each operation. Training was performed for 250 epochs and the model achieved a dice coefficient of 0.6004 on the validation set. The trained model was afterwards used to segment full-size 3520×1920 pixel images. Post-processing was performed on the binary prediction output from the U-Net to split any touching cells. A Gaussian filter was used to smooth a computed distance image, followed by locating local maximum peaks. The peaks were used as seed points for a random walker algorithm creating a label image with a unique label for each cell. A connectivity map in 25 µm isotropic resolution was downloaded from the Allen Brain Map data portal (ID 511971714). The connectivity map was resampled to 20 µm and aligned to the LSFM brain atlas space. *In situ* hybridization maps in 100 µm isotropic resolution were likewise downloaded from the ABS data portal (IDs 326, 978, 1056, 73615562) and aligned to the atlas space.

### Statistical analysis

For TH expression data, unpaired *t*-tests were performed on the accumulated unmixed voxel intensities within individual brain regions. The CCFv3 annotation volume contains 672 unique region labels; however for simplicity, smaller brain regions were merged together (respecting the hierarchical structure of the CCFv3 annotations), yielding a final number of 276 brain regions used for statistical analysis. Due to the large number of statistical tests, Benjamini–Hochberg FDR correction was performed on the calculated *P*-values. Additionally, voxel-based statistics were calculated for the TH expressions with unmixed voxel intensities being extracted for all samples and an unpaired *t*-test applied at each voxel location. The resulting significance level was noted and used for visualization. Unpaired *t*-tests were performed on counts of midbrain TH+ cells. Rotarod data were evaluated by a two-way ANOVA (treatment×time) with Dunnet's post-hoc test.

## Supplementary Material

Supplementary information
