# Supplementary material for: Quantitative whole-brain 3D imaging of tyrosine hydroxylase-labeled neuron architecture in the mouse MPTP model of Parkinson's disease
Source: Dis Model Mech. 2019 Nov 22;12(11):dmm042200. doi: 10.1242/dmm.042200 (PMC6899010; doi:10.1242/dmm.042200)
Supplement: Supplementary information [file dmm-12-042200-s1.pdf]

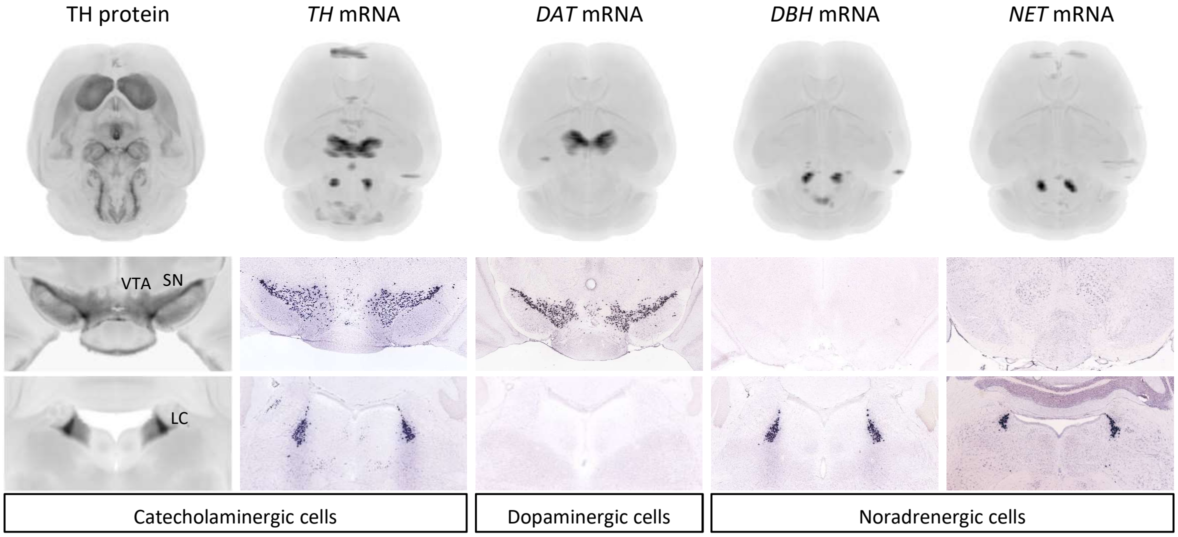

Figure S1. Phenotyping of dopaminergic and noradrenergic brain areas using *in situ* mRNA hybridization data from Allen Mouse Brain Atlas. *Tyrosine hydroxylase (TH) protein expression:* Virtual horizontal section showing average TH protein expression in vehicle control mice (3D imaging data). *mRNA expression markers*: In situ hybridization data imported from Allen Mouse Brain Atlas and mapped into the same spatial reference map as for TH protein expression. Upper panels: Horizontal brain sections showing mRNA expression of TH, dopamine transporter (DAT), dopamine beta-hydroxylase (DBT) and noradrenaline/norepinephrine transporter (NET), respectively. Lower panels: Close-up on corresponding mouse midbrain coronal sections showing in situ mRNA expression of TH, DAT, DBH and NET at the level of the midbrain and hindbrain. Abbreviations: LC, locus coeruleus; SN, substantia nigra; VTA, ventral tegmental area. Data from the Allen Mouse Brain Atlas**:** TH (AIBS_ID: 1026); DAT (*Slc6a3*, AIBS_ID: 978); DBH (AIBS_ID: 326); NET (*Slc6a2*, AIBS_ID: 511971714).

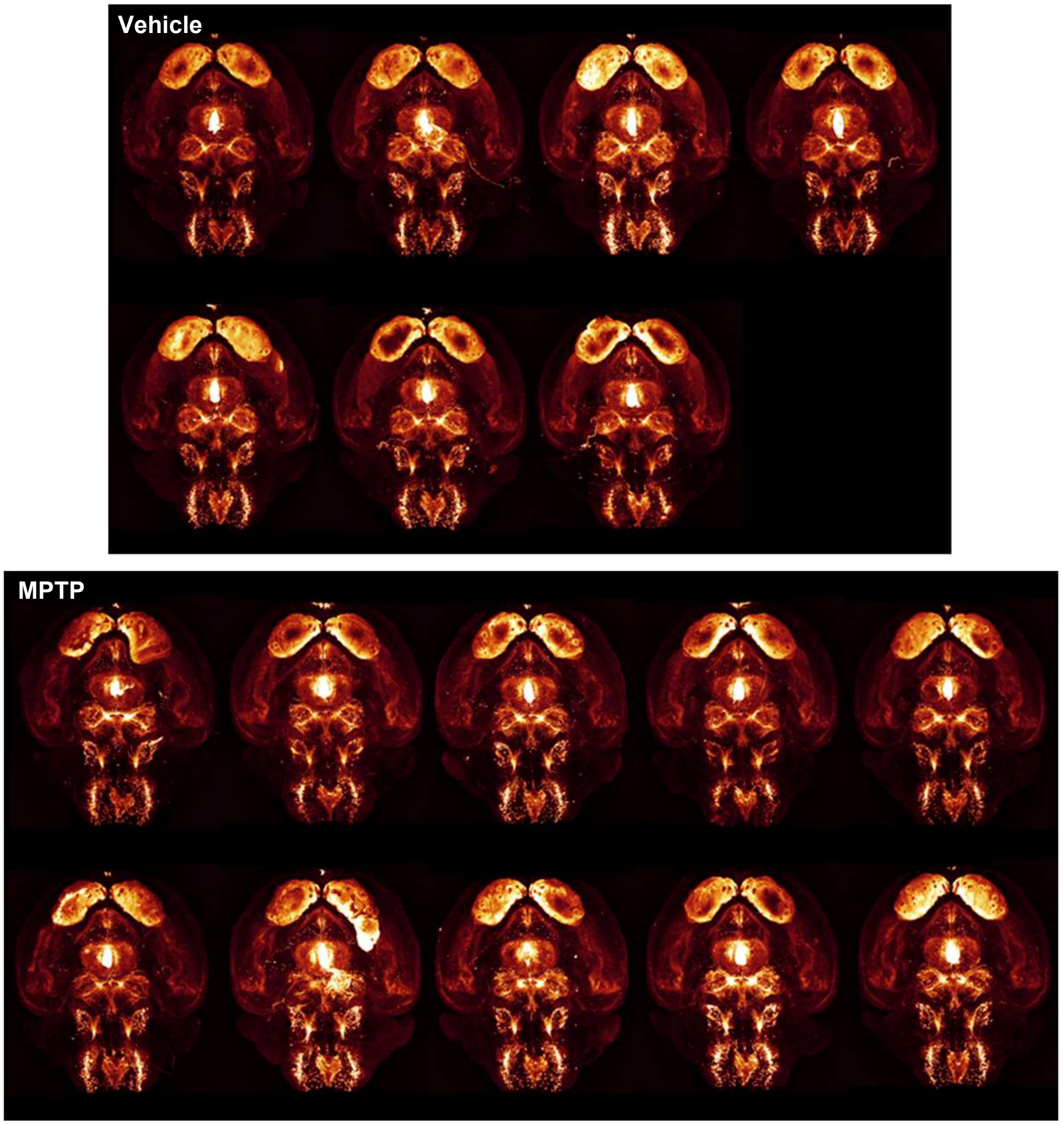

Figure S**2.** Scan of individual tyrosine hydroxylase-stained whole brains. Vehicle control mice (upper panel, n=7) and MPTP-dosed (lower panel, n=10).

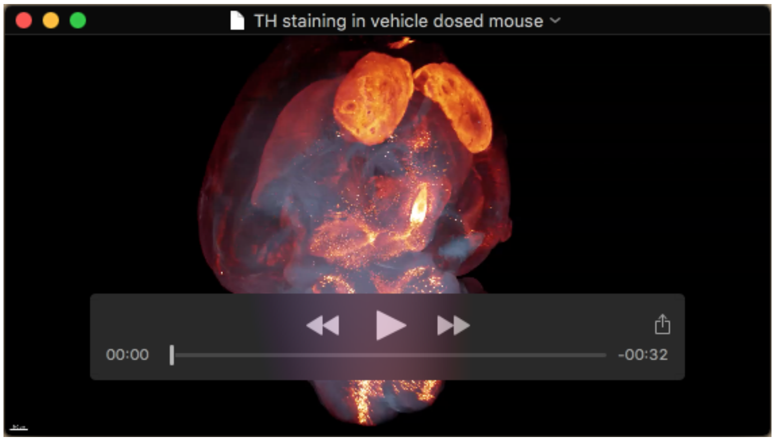

Movie 1. Whole-brain imaging of tyrosine hydroxylase staining in representative vehicle-dosed mouse.

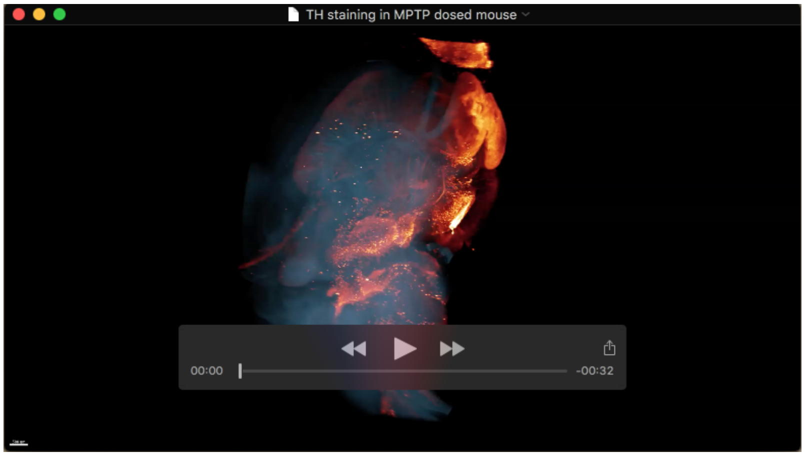

Movie 2. Whole-brain imaging of tyrosine hydroxylase staining in representative MPTP mouse.

Disease Models & Mechanisms • Supplementary information

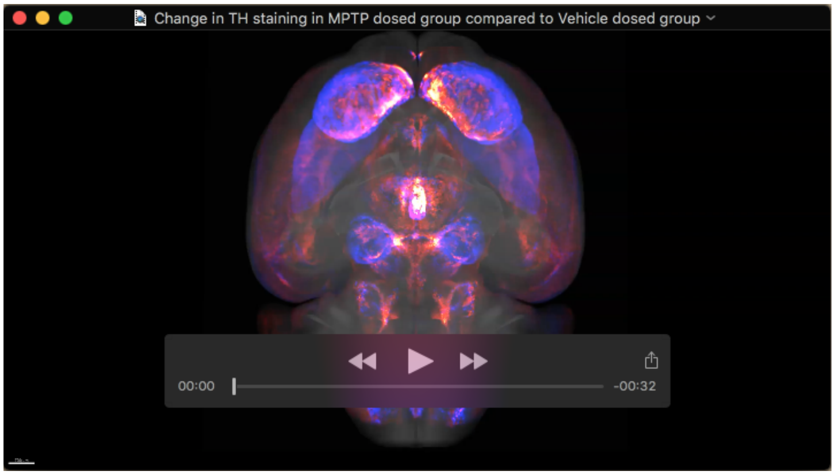

Movie 3. Group-wise comparison of average whole-brain tyrosine hydroxylase expression. MPTP mouse brain regions with significantly altered average tyrosine hydroxylase signal intensity are delineated in blue (downregulation) or red (upregulation).

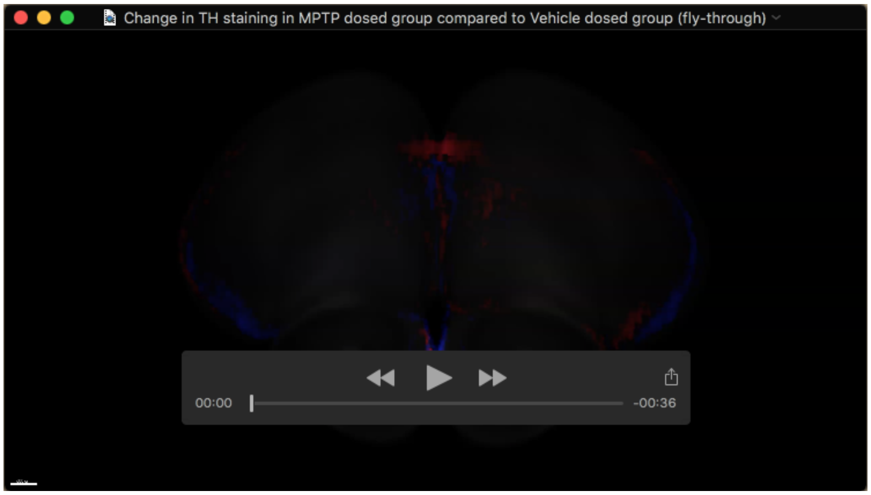

Movie 4. Fly-through visualization of changes in TH staining intensity in average MPTP mouse brain compared to average vehicle mouse control brain. MPTP mouse brain regions with significantly altered average tyrosine hydroxylase signal intensity are delineated in blue (downregulation) or red (upregulation).

Disease Models & Mechanisms • Supplementary information

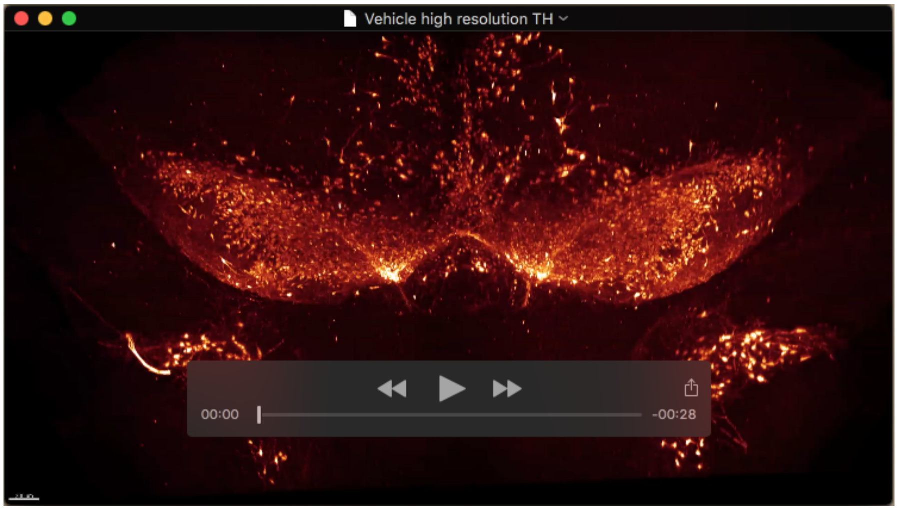

Movie 5. High-resolution imaging of midbrain tyrosine hydroxylase-positive cells in representative vehicle-dosed mouse.

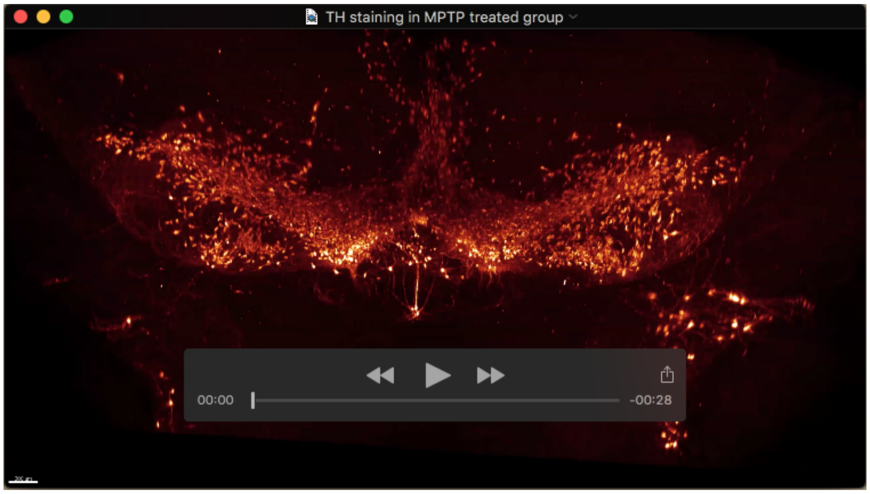

Movie 6. High-resolution whole-brain imaging of midbrain tyrosine hydroxylase-positive cells in representative MPTP mouse.

Table S1. Raw data for all brain regions

Click here to Download Table S1

Disease Models & Mechanisms • Supplementary information
